# Comparative virulence and antimicrobial resistance distribution of *Streptococcus suis* isolates obtained from the United States

**DOI:** 10.3389/fmicb.2022.1043529

**Published:** 2022-11-10

**Authors:** Tracy L. Nicholson, Darrell O. Bayles

**Affiliations:** National Animal Disease Center, Agricultural Research Service (USDA), Ames, IA, United States

**Keywords:** *Streptococcus suis*, whole-genome sequencing, comparative genomics, virulence, mobile genetic elements, antimicrobial resistance, swine

## Abstract

*Streptococcus suis* is a zoonotic bacterial swine pathogen causing substantial economic and health burdens to the pork industry worldwide. Most *S. suis* genome sequences available in public databases are from isolates obtained outside the United States. We sequenced the genomes of 106 *S. suis* isolates from the U.S. and analyzed them to identify their potential to function as zoonotic agents and/or reservoirs for antimicrobial resistance (AMR) dissemination. The objective of this study was to evaluate the genetic diversity of *S. suis* isolates obtained within the U.S., for the purpose of screening for genomic elements encoding AMR and any factors that could increase or contribute to the capacity of *S. suis* to transmit, colonize, and/or cause disease in humans. Forty-six sequence types (STs) were identified with ST28 observed as the most prevalent, followed by ST87. Of the 23 different serotypes identified, serotype 2 was the most prevalent, followed by serotype 8 and 3. Of the virulence genes analyzed, the highest nucleotide diversity was observed in *sadP*, *mrp*, and *ofs*. Tetracycline resistance was the most prevalent phenotypic antimicrobial resistance observed followed by macrolide-lincosamide-streptogramin B (MLSB) resistance. Numerous AMR elements were identified, many located within MGE sequences, with the highest frequency observed for *ble*, *tetO* and *ermB*. No genes encoding factors known to contribute to the transmission, colonization, and/or causation of disease in humans were identified in any of the *S. suis* genomes in this study. This includes the 89 K pathogenicity island carried by the virulent *S. suis* isolates responsible for human infections. Collectively, the data reported here provide a comprehensive evaluation of the genetic diversity among U.S. *S. suis* isolates. This study also serves as a baseline for determining any potential risks associated with occupational exposure to these bacteria, while also providing data needed to address public health concerns.

## Introduction

*Streptococcus suis* is a bacterial swine pathogen that causes significant economic losses to the swine industry worldwide (Feng et al., 2014; Segura et al., 2014a,b). *S. suis* causes a wide variety of clinical diseases in pigs including pneumonia, endocarditis, septicemia, and meningitis (Votsch et al., 2018). In addition, and perhaps more notably, *S. suis* is a zoonotic pathogen capable of causing invasive diseases in humans, mainly arthritis, meningitis, as well as streptococcal toxic shock-like syndrome (STSS), which can lead to rapid death (Wertheim et al., 2009; Feng et al., 2014; Segura et al., 2014a,b). In fact, *S. suis* is the most frequently diagnosed cause of adult bacterial meningitis in Vietnam (Mai et al., 2008; Wertheim et al., 2009; Feng et al., 2014). While human infections can be sporadic, most human infections are thought to be acquired from penetrating injuries associated with occupational exposure or consumption of raw or undercooked pork products. The predominance of either route of infection tends to vary with geographic location. For example, in Asia, consumption of raw or undercooked pork products has been attributed to the majority of human clinical cases (Gottschalk et al., 2007; Dutkiewicz et al., 2018). In contrast, human *S. suis* infection is considered a swine-related occupational disease in western countries (Gottschalk et al., 2007; Dutkiewicz et al., 2018).

Colonization and virulence mechanisms used by *S. suis* are not comprehensively characterized (Fittipaldi et al., 2012; Segura et al., 2017). Studies addressing specific virulence mechanisms used by *S. suis* have been confounded because different isolates cause a spectrum of disease outcomes ranging from lethal systemic disease to asymptomatic carriage (Segura et al., 2017; Votsch et al., 2018). The specific factors generally regarded as the most important to virulence are the capsular polysaccharide (CPS), muramidase-released protein (*mrp*), extracellular protein factor (*epf*), and suilysin (*sly*; Fittipaldi et al., 2012; Segura et al., 2014a,b, 2017). However, none of these factors independently correlate with the ability to cause invasive systemic disease and therefore virulence is thought to be multifactorial (Fittipaldi et al., 2012; Segura et al., 2017). Moreover, invasive clinical isolates obtained from both people and pigs often do not harbor all of these factors (Fittipaldi et al., 2012; Feng et al., 2014; Segura et al., 2014a, 2017).

*Streptococcus suis* is considered to have an incompletely defined (open) pan-genome since the number of unique or accessory genes increases and the number of conserved genes decreases as more genomes are sequenced (Wang et al., 2011; Shelyakin et al., 2019; Guo et al., 2021). The open pan-genome is reflected in the high diversity among *S. suis* isolates, and high rates of horizontal gene transfer by mobile genetic elements (MGEs) have been reported (Holden et al., 2009; Wang et al., 2011; Weinert et al., 2015; Willemse et al., 2016; Guo et al., 2021). MGEs harboring virulence factors or antimicrobial resistance (AMR) genes are ubiquitous in bacteria and are the single most significant driver of gene transfer enabling bacteria to evolve and become more pathogenic. The 89 K pathogenicity island (89 K PAI) carried by Chinese epidemic strains is an example of a *S. suis* MGE harboring factors contributing to a highly invasive phenotype (Chen et al., 2007; Schmid et al., 2011). AMR has been extensively reported for *S. suis* isolates worldwide and many of the genes conferring AMR are passenger genes carried on MGEs (Palmieri et al., 2011a; Gurung et al., 2015; Huang et al., 2016b, 2018; Pan et al., 2019; Aradanas et al., 2021; Dechene-Tempier et al., 2021; Hadjirin et al., 2021; Ma et al., 2021). For this reason, *S. suis* is regarded a reservoir for AMR that can be easily transferred to other bacterial commensals and pathogens (Palmieri et al., 2011b; Varela et al., 2013).

Some recent studies have undertaken whole-genome sequencing (WGS) in combination with comparative genomic approaches to evaluate the genomic diversity and AMR elements harbored by *S. suis* isolates (Weinert et al., 2015; Wileman et al., 2019; Hadjirin et al., 2021; Estrada et al., 2022). The majority of these genomic sequences needed to evaluate potential risks attributed to the capacity of *S. suis* isolates to successfully function as zoonotic agents and/or reservoirs for AMR dissemination, is derived from isolates obtained outside the U. S (Weinert et al., 2015; Wileman et al., 2019; Hadjirin et al., 2021; Estrada et al., 2022). The goals of the current study were to fill this gap by utilizing whole-genome sequencing (WGS) analysis to evaluate the sequence type (ST) and serotype distribution of *S. suis* isolates obtained within the U.S., screen genomes for genomic elements encoding AMR, determine whether or not identified AMR genes are located within MGEs, and screen for genomic elements encoding factors known to increase or contribute to the capacity of *S. suis* to transmit, colonize, and/or cause disease in humans.

## Materials and methods

### *Streptococcus suis* isolates and culture conditions

A total of 106 *S. suis* isolates obtained from swine samples collected within the US and submitted to the University of Minnesota Veterinary Diagnostic Laboratory between 2015 and 2017 were selected for the project (Supplementary Table 1). Frozen stocks (−80°C in 30% glycerol) of *S. suis* isolates were streaked onto tryptic soy agar containing 5% sheep blood (Becton, Dickinson and Co. Franklin Lakes, NJ) and grown overnight aerobically at 37°C with 5% CO2. Single colonies were used to inoculate Todd-Hewitt broth (Thermo Fisher Scientific Inc., Waltham, MA) supplemented with 0.2% yeast extract (MilliporeSigma, St. Louis, MO) and 5% filtered heat-inactivated horse serum (MilliporeSigma, St. Louis, MO). Broth cultures were grown aerobically at 37°C overnight in a shaking incubator (250 rpm).

### Whole-genome sequencing, assembly, and annotation

Genomic DNA was extracted using a MasterPure Gram Positive DNA Purification Kit (Lucigen Corporation, Middleton, WI) with the following modifications to the manufacturer’s instructions to prevent degradation. One mL from overnight broth cultures was pelleted by centrifugation (5,000× *g* for 5 min), the supernatant was removed, and the pelleted cells were resuspended in 300 μl lysis buffer consisting of 2% SDS (Thermo Fisher Scientific Inc., Waltham, MA), 0.25 M EDTA (Thermo Fisher Scientific Inc., Waltham, MA), and 30% Proteinase K (Roche, Mannheim, Germany). The suspension was incubated for 3 h at 55°C. 300 μl of Gram Positive Lysis solution from the MasterPure Gram Positive DNA Purification Kit was added and the suspension was incubated for 30 min at 70°C. Samples were then placed on ice for 5 min, 350 μl of MPC Protein Precipitation Reagent from the MasterPure Gram Positive DNA Purification Kit was added and the DNA precipitation instructions provided by the manufacturer’s protocol were followed. DNA was quantified using a Qubit Fluorometer (Thermo Fisher Scientific, Waltham, MA) and the integrity was further checked using 1% agarose gel electrophoresis.

Illumina HiSeq data was obtained from a library created using the NEBNext Ultra II DNA Library Prep Kit (New England Biolabs, Ipswich, MA) sequenced on a HiSeq 3000 instrument generating 2 × 150 bp paired-end reads. Sequencing reads were assessed for quality using FastQC.^1^ Reads were randomly subsampled using seqtk^2^ to target a genome coverage of 150X based on an average expected genome length of 2.1 Mbases. Sequence data was assembled using MIRA v. 4.9.6 (Chevreux et al., 1999).^3^ The average coverage obtained for each isolate is listed in Supplementary Table 1. To be retained in an assembly, contigs were required to be >1,500 bp in length and have a coverage of >66% of the average coverage for the genome. The assembly tool identified repetitive elements that were required to have a contig length >2,000 bp to remain in the assembly. Unless specified otherwise, default parameters were used for all software. Final annotations were completed using NCBI’s Prokaryotic Genome Annotation Pipeline (PGAP) v 4.11 (Tatusova et al., 2016). Accession numbers and genome statistics are summarized in Supplementary Table 1.

### Comparative genomic analysis

MLST and serotype was determined *in silico* using the automated pipeline developed by Athey et al. (2016), which uses short-read sequencing data to: assign sequence types (STs) based on the MLST scheme developed by King et al. (2002), determine *S. suis* serotypes, and confirm isolate as *S. suis* based on nucleotide sequence of the *recN* gene. PCR and Sanger sequencing were subsequently utilized to determine ST for all remaining isolates with an unknown and/or uncertain ST. The ST could not be determined for two isolates (40422 and 40430). The serotyping results were further analyzed *in silico* based on the PCR typing schemes described by Liu et al. (2013) for the classical serotypes and by Qiu et al. (2016) for the Novel CPS Loci (NCL) serotypes to confirm the identification and locate the capsule loci within genome assemblies. Clonal complexes were identified by goeBURST analysis (Francisco et al., 2009) using the set of *S. suis* MLST profiles downloaded from PubMLST.^4^ This set contained 1690 unique profiles. Using previously reported stringency criteria, a clonal complex (CC) was defined as a group of STs comprised of at least six identical alleles and containing at least three STs (Single Locus Variant (SLV) clustering) (Wang et al., 2015; Scherrer et al., 2020). STs that did not fall into any group were classified as singlets and STs that grouped with only one other ST were classified as doublets. A group of three STs that differ from each other by a single locus but lack other connections were not assigned a CC and classified as having no clear founder.

Potential virulence-associated genes, which were included in a comprehensive review (Fittipaldi et al., 2012), were identified by BLASTN searches and the percent identity for each gene was determined for each isolate relative to the P1/7 orthologue with the following exceptions: *epf, hylA*, *ofs, revS*, *stp*, *vraR* and *vraS*. For these exceptions, a reference gene sequence from a different strain was chosen because functional characterization had been previously reported from that strain or the corresponding gene is annotated as pseudogene in P1/7. Nucleotide percentage identity for each gene was converted into a distance matrix heatmap and clustered by means of complete hierarchical clustering based on Pearson correlation distance for both genes and isolates using MeV 4.8.1 (Saeed et al., 2006). The Virulence Factor Data Base (Liu et al., 2022) that includes genes associated with experimentally verified virulence factors was employed to search for CDSs encoding virulence-associated factors with parameters of 80% identity 60% coverage results.

### Phenotypic and genomic AMR analysis

Phenotypic antibiotic resistance was determined using the broth microdilution method by National Veterinary Services Laboratories (Ames, IA) following standard operating procedures. Minimum inhibitory concentrations (MICs) were determined for each isolate using the Trek BOPO7F plate (Thermo Fisher Scientific Inc., Oakwood Village, OH) with *Streptococcus pneumoniae* ATCC 49619 and *Mannheimia haemolytica* ATCC 33369 (ATCC, Manassas, VA) serving as the quality control strains. MICs were evaluated in accordance with Clinical Laboratory Standards Institute (CLSI) recommendations based on the VET08 and M100 standards for resistance interpretations (Supplementary Table 2; Clinical and Laboratory Standards Institute, 2018, 2020). Abricate (Seeman T, Abricate, Github^5^) was used to identify antimicrobial resistance genes using the AMR gene databases from the Comprehensive Antibiotic Resistance Database (CARD; Jia et al., 2017), ResFinder (Center for Genomic Epidemiology; Zankari et al., 2012) and the NCBI Bacterial Antimicrobial Resistance Reference Gene Database (BioProject Accession PRJNA313047), which was downloaded in April 2019. A minimum percent identity threshold of 80% was used to identify AMR genes in the assembled genomes.

### Identification and classification of MGEs

Whole-genome sequence data for all isolates in this study, as well as genome sequence data from previously reported *S. suis* isolates (Nicholson et al., 2020), were screened *in silico* for MGEs. Identification and typing of plasmids was performed by using PlasmidFinder (Carattoli et al., 2014; accessed 17 February 2022), with parameters of 80% identity 50% covered length. Identification of MGEs was conducted by employing MGEfinder v1.0.6 (Durrant et al., 2020). PGAP annotations were used to identify CDSs contained within each representative MGE sequence that encode conjugation VirB4 proteins, MOB relaxases, serine integrases PF00239 (resolvase with N-terminal domain), PF07508 (Recombinase), and PF13408 (recombinase zinc beta-ribbon domain), tyrosine integrases PF00589 (site-specific prophage integrase), PF02899 (prophage integrase with N-terminal SAM-like domain), PF09003 (bacteriophage lambda integrase with N-terminal domain), TIGR02225 (tyrosine recombinase XerD), TIGR02224 (tyrosine recombinase XerC), and PF13102 (prophage integrase SAM-like domain), and Group II introns TIGR04416 (group II intron reverse transcriptase maturase). *in silico* PCR was used to screen genome sequences in this study, as well as previously reported *S. suis* genomes (Nicholson et al., 2020), employing primer sets described by Schmid et al. (2011). All representative MGE sequences were classified into categories in the following order. If a MGE sequence was predicted as a Prophage by PHASTER (Arndt et al., 2016), then it was assigned to the “Prophage” category. If a MGE sequence was predicted as an IS Element by ISEScan (Xie and Tang, 2017), then it was assigned to the “IS Element” category. If a MGE sequence contained a conjugation VirB4, a MOB relaxase, and an integrase or transposase at the boundary of the element, then it was assigned to the “ICE” category. Putative MGEs were annotated as IMEs when no VirB4 CDS was present and when an integrase CDS was found in the vicinity of a relaxase CDS. If a MGE sequence was not classified as an IME and contained a transposase and the sequence length was between 10 and 30 kb, then it was assigned to the “transposon” category. If a MGE sequence contained a Group II intron and did not contain any transposases, and the sequence length was less than 10 kb, then it was assigned to the “Group II intron” category. All remaining unclassified MGEs were then divided into two groups based on their sizes: unclassed genomic islands (>10 kb), or islets (<10 kb). It should be noted that these categories were NOT forced to be mutually exclusive, since these elements share some overlap. For example, many predicted phages carry serine recombinases.

### Data availability statement

The Whole Genome Shotgun project has been deposited at DDBJ/ENA/GenBank under the BioProject accession number PRJNA604583 and Sequence Read Archive (SRA) study number SRP341913. Detailed information regarding BioSample, GenBank accession numbers, and SRA accession numbers are provided in Supplementary Table 1.

## Results

### Sequence type and serotype distribution

Serotyping and multilocus sequence typing (MLST) are the most common methods used to differentiate *S. suis* isolates. A total of forty-six different sequence types (STs) were identified with ST28 observed as the most prevalent, accounting for 21% (*n* = 22), followed by ST87 (10%, *n* = 11), ST94 (6%, *n* = 6), and ST961 (5%, *n* = 5; Table 1). STs were subsequently assigned to clonal complexes (CCs) and seventeen CCs were identified (Table 1). The distribution of *S. suis* isolates in relation to ST revealed CC28 was the most prevalent, accounting for 29% (*n* = 31), followed by CC87 (11%, *n* = 12), and CC94 (7%, *n* = 7; Table 1).

**Table 1:** Prevalence of clonal complexes (CC) and sequence types (ST) among *Streptococcus suis* isolates.

CC	Number (%)^a^	ST	Number (%)^a^
CC28	31 (29)	28	22 (21)
		961	5 (5)
		1,180	4 (4)
CC87	12 (11)	87	11 (10)
		1,181	1 (<1)
CC94	7 (7)	94	6 (6)
		966	1 (<1)
CC108	5 (5)	108	4 (4)
		977	1 (<1)
CC373	5 (5)	373	4 (4)
		1,199	1 (<1)
CC839	4 (4)	89	1 (<1)
		119	2 (2)
		1,196	1 (<1)
CC1198	3 (3)	790	2 (2)
		1,198	1 (<1)
CC1	2 (2)	1	2 (2)
CC17	2 (2)	17	2 (2)
CC27	2 (2)	27	2 (2)
CC969	2 (2)	969	2 (2)
CC76	1 (<1)	76	1 (<1)
CC225	1 (<1)	225	1 (<1)
CC968	1 (<1)	968	1 (<1)
CC1175	1 (<1)	1,197	1 (<1)
CC1200	1 (<1)	1,200	1 (<1)
CC1290	1 (<1)	778	1 (<1)
None^c^	24 (23)	54	3 (3)
		985	1 (<1)
		1,194	1 (<1)
		1,195	1 (<1)
		1,201	1 (<1)
		1,202	1 (<1)
		1,203	1 (<1)
		1,204	1 (<1)
		1,205	1 (<1)
		1,206	1 (<1)
		1,207	1 (<1)
		1,208	1 (<1)
		1,209	1 (<1)
		1,210	1 (<1)
		1,211	1 (<1)
		1,212	1 (<1)
		1,306	1 (<1)
		1,311	1 (<1)
		1,312	1 (<1)
		1,313	1 (<1)
		NF^b^	2 (2)

^a^Number of isolates harboring indicated CC or ST (percentage of isolates). ^b^ST could not be determined using either *in silico* or PCR combined with Sanger sequencing methods. ^c^STs defined as singletons, doublet, or having no clear founder were classified as none.

Molecular serotyping of the isolates identified 23 different serotypes with serotype 2 observed as the most prevalent, accounting for 26% (*n* = 28), followed by serotype 3 (11%, *n* = 12), and 8 (11%, *n* = 12; Table 2). Ten isolates (9%) harbored a capsule type in which the capsule genes were present, but locus was either incomplete or could not be assigned because the genes did not match any known capsule types and were therefore classified as undetermined (UND; Table 2). Other serotypes were detected quite rarely, including serotypes 10, 14, 15, 16, and 23 (two isolates each, 2%) and serotypes 11, 2, 17, 18, 19, 27, 28, 30, and 31 (one isolate each, <1%; Table 2).

**Table 2:** Prevalence of serotypes among *S. suis* isolates.

Serotype	Number (%)^a^
2	28 (26)
3	12 (11)
8	12 (11)
UND^b^	10 (9)
7	9 (8)
9	5 (5)
4	4 (4)
5	4 (4)
1/2	3 (3)
10	2 (2)
14	2 (2)
15	2 (2)
16	2 (2)
23	2 (2)
11	1 (<1)
12	1 (<1)
17	1 (<1)
18	1 (<1)
19	1 (<1)
27	1 (<1)
28	1 (<1)
30	1 (<1)
31	1 (<1)

^a^Number of isolates harboring indicated serotype (percentage of isolates). ^b^UND (undetermined); serotypes were classified as undetermined when capsule locus was either incomplete or did not match any known serotypes.

### AMR distribution

Phenotypic antimicrobial resistance was determined and 53 isolates (50%) were resistant to three out of the eight antibiotic classes tested (Table 3). The highest frequencies of resistance were observed for tetracycline (97%, *n* = 103), macrolide/lincosamide/streptogramin (MLSb; 72%, *n* = 76), and sulfonamide (46%, *n* = 49) antibiotic classes (Table 3). In contrast, the lowest frequencies of resistance were observed for aminoglycoside (9%, *n* = 9), β-lactam (8%, *n* = 8), and fluroquinolone (2%, *n* = 2) antibiotic classes (Table 3). No isolates were found to be phenotypically resistant to the following specifically tested antibiotics: ampicillin, ceftiofur, florfenicol, or trimethoprim/sulphamethoxazole (Table 3).

**Table 3:** Phenotypic AMR prevalence among *S. suis* isolates.

Antibiotic class	Antibiotic	Number (%)^a^
Aminoglycoside	Gentamicin	2 (2%)
	Neomycin	9 (9%)
Beta-Lactam	Ampicillin	0 (0%)
	Penicillin	8 (8%)
	Ceftiofur	0 (0%)
Fluroquinolone	Enrofloxacin	2 (2%)
Macrolide/Lincosamide/Streptogramin (MLSb)	Clindamycin	76 (72%)
	Tilmicosin	71 (67%)
	Tulathromycin	69 (65%)
	Tylosin	70 (66%)
Phenicol	Florfenicol	0 (0%)
Sulfonamide	Sulphadimethoxine	49 (46%)
	Trimethoprim/Sulphamethoxazole	0 (0%)
Pleuromutilin	Tiamulin	22 (21%)
Tetracycline	Tetracycline	103 (97%)

^a^Number of isolates resistant to indicated antibiotic (percentage of isolates).

**Figure 1:**
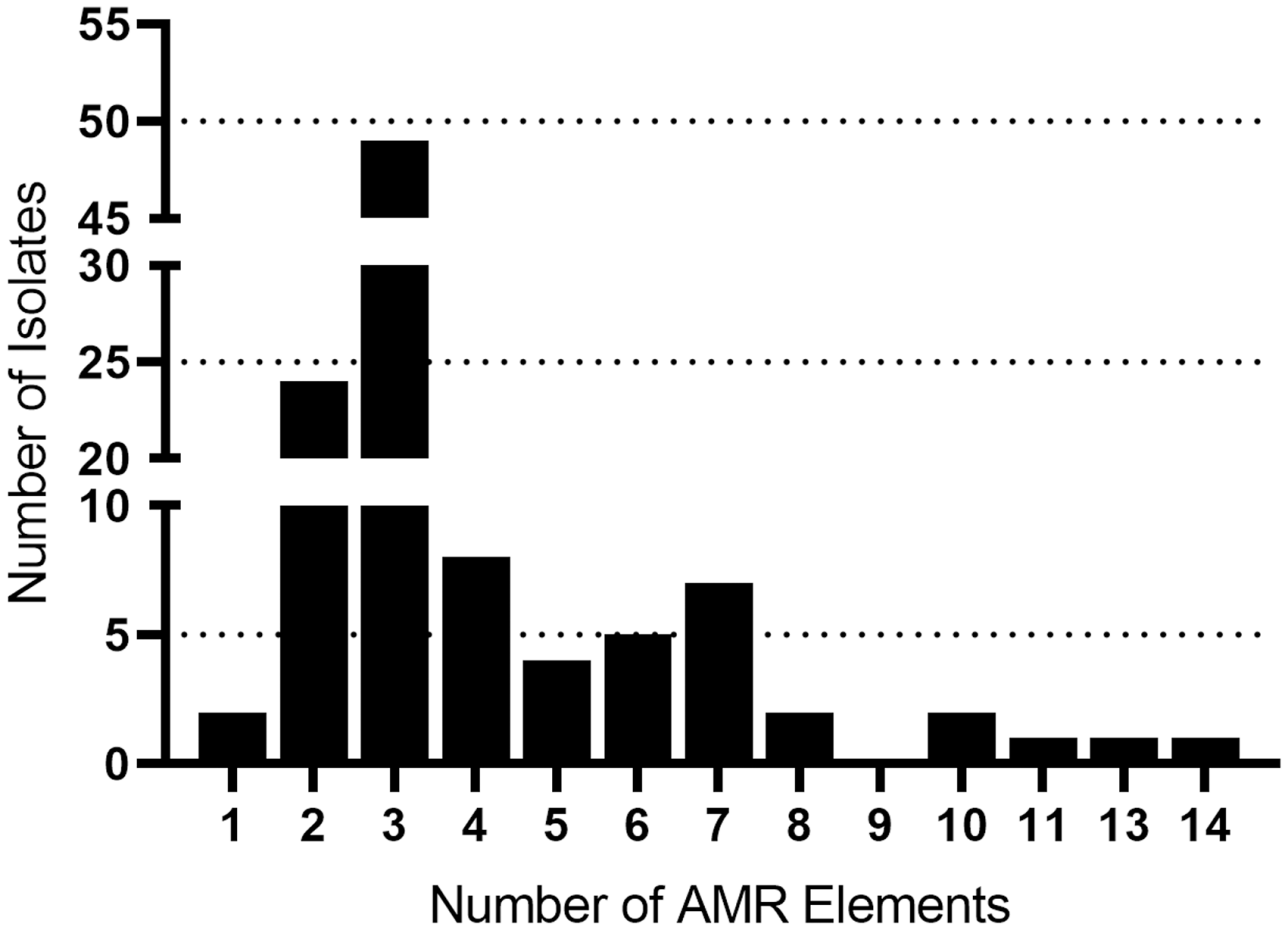
AMR gene frequency among *Streptococcus suis* isolates. The x axis indicates the number of AMR genes harbored by a single isolate. The y axis indicates the number of isolates identified harboring each number of AMR genes.

Draft genomes were screened for chromosomal mutations and genes conferring antimicrobial resistance and a total of 401 AMR elements were found (Table 4; Supplementary Table 2). The number of AMR elements harbored by an individual isolate ranged from one to fourteen (Figure 1; Supplementary Table 2). Isolates 40420 and 40458 each harbored one AMR element, while fourteen were identified in isolate 40422. Numerous isolates harbored two or more AMR elements (Figure 1; Supplementary Table 2). Specifically, 104 isolates (98%) harbored two AMR or more elements and 80 isolates (75%) harbored three or more AMR elements (Figure 1; Supplementary Table 2).

**Table 4:** AMR elements identified.

Resistance genes	Number (%)^a^
Aminoglycoside Resistance	
*aadD1*	1 (<1%)
*ant(9)-Ia*	13 (12%)
*aph(3′)-IIIa*	6 (6%)
*aac(6′)-aph(2″)*	1 (<1%)
*aad9*	2 (2%)
*ant(6)-Ia*	12 (11%)
*ant(6)-Ib*	1 (<1%)
*aph(2″)-IIIa*	1 (<1%)
*aph(3′)-IIIa*	6 (6%)
Glycopeptide Resistance	
*ble*	106 (100%)
Macrolide/Lincosamide/Streptogramin (MLSb) Resistance	
*lnuA*	2 (2%)
*lnuC*	1 (<1%)
*lnuD*	3 (3%)
*lsaE*	15 (14%)
*lnuB*	15 (14%)
*erm47*	3 (3%)
*ermB*	71 (67%)
*vgb*	3 (3%)
*vat*	3 (3%)
Pleuromutilin Resistance	
*lsaE*	15 (14%)
Nucleoside Resistance	
*sat4*	5 (5%)
Tetracycline Resistance	
*tet(40)*	2 (2%)
*tet(44)*	1 (<1%)
*tetL*	4 (4%)
*tetM*	7 (7%)
*tetO*	91 (86%)
*tet(O/W/32/O)*	4 (4%)
*tetT*	3 (3%)
*tetW*	1 (<1%)

^a^Number of isolates harboring indicated AMR gene or element including partial CDSs (percentage of isolates).

The most prevalent AMR element found was *ble* (100%, *n* = 106) followed by *tetO* (86%, *n* = 91), *ermB* (67%, *n* = 71), *lsaE* (14%, *n* = 15), *lnuB* (14%, *n* = 15), *ant(9)-Ia* (12%, *n* = 13), and *ant(6)-Ia* (11%, *n* = 12; Table 4; Supplementary Table 2). Additionally, the *lsaE* and *lnuB* genes were located adjacent to each other in every isolate harboring the *lsaE* and *lnuB* genes. Similarly, *ant(6)-Ia* was co-located next to *ant(9)-Ia* in every isolate harboring *ant(6)-Ia* (Supplementary Table 2).

**Figure 2:**
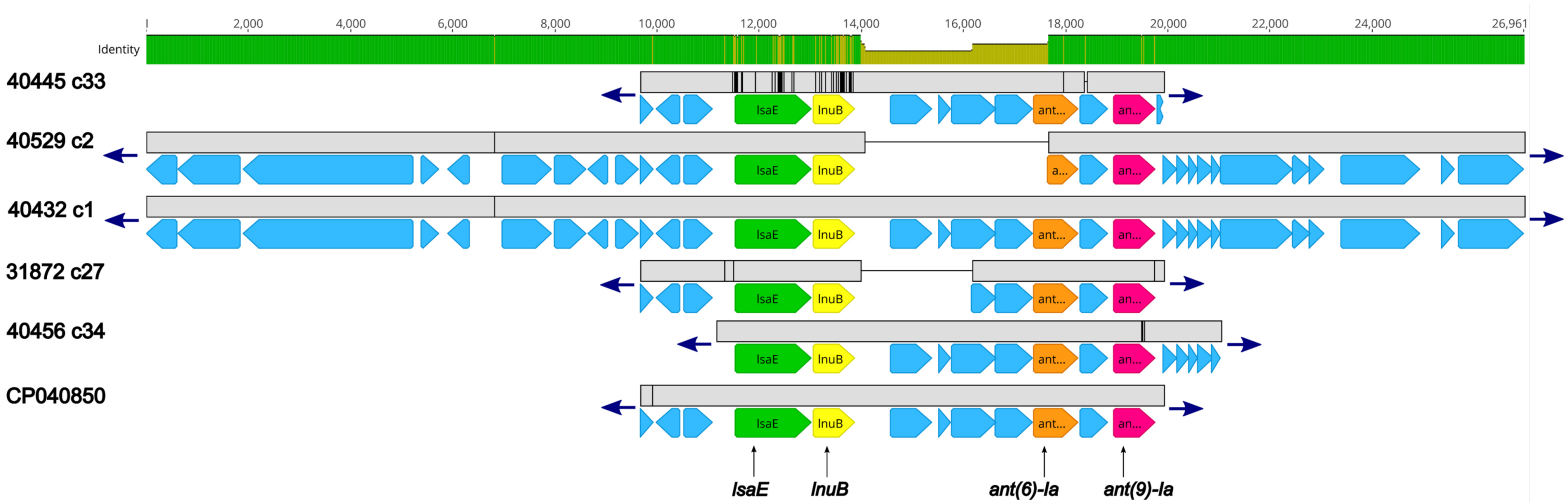
Genomic organization and alignment of the region containing the *lsaE*, *lnuB, ant(6)-Ia,* and *ant(9)-Ia* genes. The *S. suis* isolate and contig number (c) for each sequence, along with the accession number for the *Enterococcus faecium* plasmid shown at right. Base pair numbers are displayed above black bar at top. Green and yellow shaded bar below depicts sequence similarity with green indicating 100% identity and yellow indicating lower identity. Grey bar in between each aligned sequence region depicts sequence similarity between each sequence with grey indicating nucleotide positions with 100% identity. Blue arrows at end of grey bars indicate contig sequence continues. AMR gene elements are listed below and indicated by green (*lsaE*), yellow (*lnuB*), orange (*ant(6)-Ia*), and pink (*ant(9)-Ia*) genes. All other CDSs are indicated by light blue. The *ant(6)-Ia* gene in 40529 c2 is a partial gene (missing N-terminus) and is flagged as predicted pseudogene by PGAP. Accession number CP040850 is a complete nucleotide sequence from an *Enterococcus faecium* strain F17E0263 plasmid (p_unnamed1) isolated from a chicken.

Further examination revealed that for five isolates (40445, 40529, 40432, 31872, and 40456) the *lsaE*, *lnuB, ant(6)-Ia,* and *ant(9)-Ia* genes were co-located in a region approximately 10 Kb in length that shared a high degree of nucleotide similarity to an *Enterococcus faecium* plasmid (accession no. CP040850; Figure 2; Supplementary Table 2). 100% sequence identity was observed for each of the *lsaE, lnuB,* and *ant(6)-Ia* genes of the *E. faecium* plasmid and isolates 31872, 40432, 40456, 40529. The *lsaE, lnuB,* and *ant(6)-Ia* genes harbored by isolate 40445 shared a lower sequence identity compared to the other isolates and the *E. faecium* plasmid with a 98.5% sequence identity for *lsaE*, a 97% sequence identity for *lnuB,* and a 99.8% sequence identity for *ant(6)-Ia* (Figure 2; Supplementary Table 2). 100% sequence identity was observed for the *ant(9)-Ia* gene of the *E. faecium* plasmid and isolates 40432, 40445, and 40529. The *ant(9)-Ia* gene harbored by isolate 40456 and 31872 shared a lower sequence identity compared to the other isolates and the *E. faecium* plasmid, with a 99.5% sequence identity for the *ant(9)-Ia* gene from isolate 31872 and a 99.6% sequence identity for the *ant(9)-Ia* gene from isolate 40456 (Figure 2; Supplementary Table 2).

**Figure 3:**
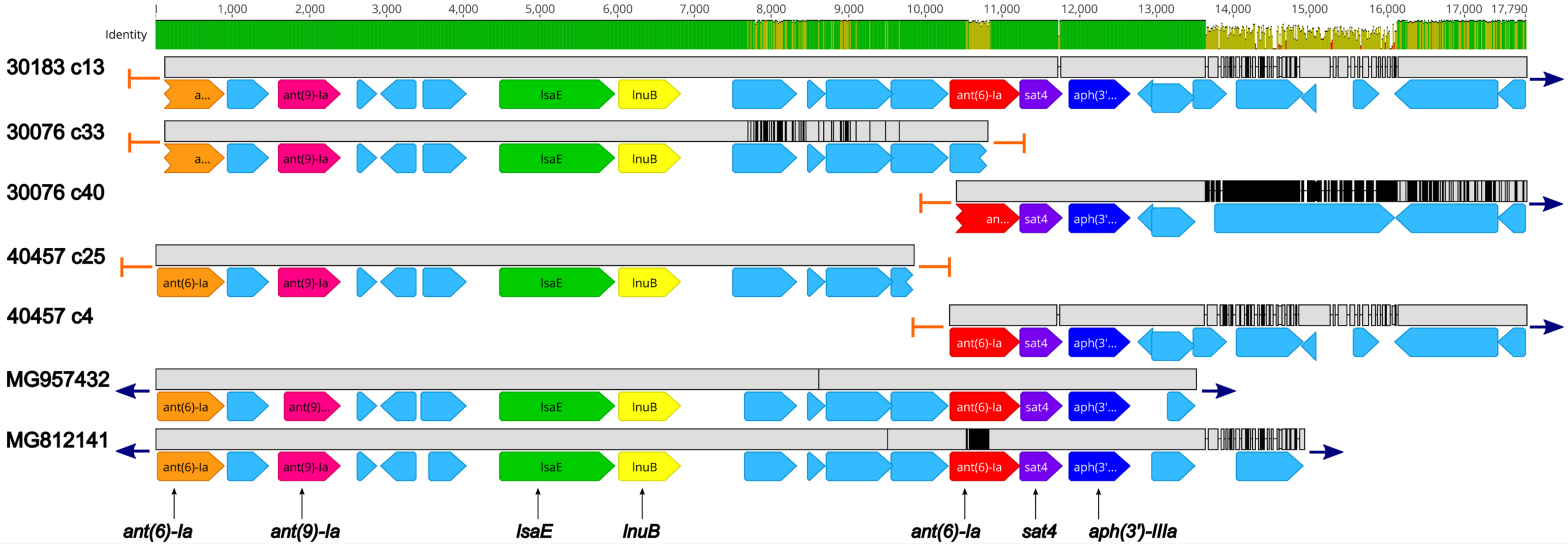
Genomic organization and alignment of the region containing the *ant(6)-Ia, sat4,* and *aph(3′)-IIIa* genes. The *S. suis* isolate and contig number (c) for each sequence, along with the accession numbers for the *Enterococcus faecium* plasmid and the *Erysipelothrix rhusiopathiae* ICE (ICCEr0106) shown at right. Base pair numbers are displayed above black bar at top. Green and yellow shaded bar below depicts sequence identity with green indicating 100% identity and yellow indicating lower identity. Grey bar in between each aligned sequence region depicts sequence similarity between each sequence with grey indicating nucleotide positions with 100% identity. Blue arrows at end of grey bars indicate contig sequence continues and orange bars indicate end of contig sequence. AMR gene elements are listed below and are indicated by orange (*ant(6)-Ia*), pink (*ant(9)-Ia*), green (*lsaE*), yellow (*lnuB*), red (*ant(6)-Ia*), purple (*sat4*), and dark blue (*aph(3′)-IIIa*). All other CDSs are indicated by light blue. Predicted incomplete CDSs are indicated by jagged ends. Accession number MG957432 is a complete nucleotide sequence from *Enterococcus faecium* strain 37BA conjugative plasmid (pEf37BA) isolated from a human. Accession number MG812141 is a complete nucleotide sequence from *Erysipelothrix rhusiopathiae* ICE (ICCEr0106).

Five isolates were found to harbor the *sat4* gene, encoding a streptothricin acetyltransferase, conferring resistance to streptothricin, a nucleoside antibiotic. The *sat4* was co-located with the aminoglycoside resistant genes *ant(6)-Ia* and *aph(3′)-IIIa* (Figure 3; Supplementary Table 2). Further analysis revealed a high degree of nucleotide similarity of an approximately 13.5 Kb region in three of the isolates (30183, 30076, and 40457) containing the *sat4* gene and two MGEs, *Enterococcus faecium* multidrug resistance conjugative plasmid (pEf37BA; accession no. MG957432) and *Erysipelothrix rhusiopathiae* integrative conjugative element (ICCEr0106; accession no. MG812141; Figure 3; Supplementary Table 2). The region includes *ant(6)-Ia*, *ant(9)-Ia, lsaE*, and *lnuB* genes upstream of the *ant(6)-Ia*, *sat4*, and *aph(3′)-IIIa* elements (Figure 3; Supplementary Table 2). In all five regions a 100% sequence identity was observed for the *ant(6)-Ia*, *ant(9)-Ia, lsaE*, *lnuB,* and *aph(3′)-IIIa* genes (Figure 3; Supplementary Table 2). Lower differences in sequence similarity were observed for the *ant(6)-Ia* (91.9–100%) and *sat4* (97.8–100%) genes located adjacent to *aph(3′)-IIIa* (Figure 3; Supplementary Table 2).

**Figure 4:**
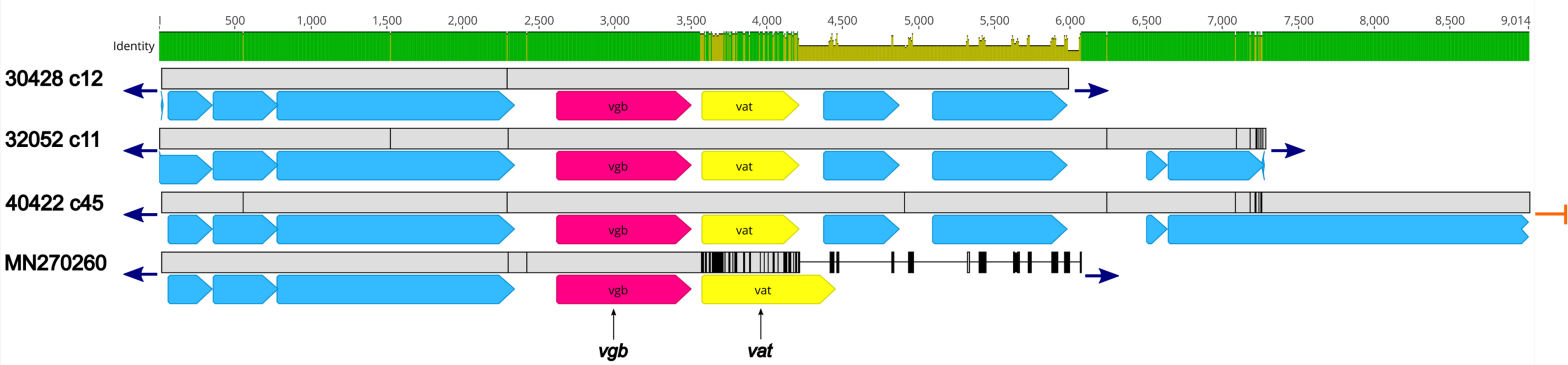
Genomic organization and alignment of the region containing the *vgb* and *vat* genes. The *S. suis* isolate and contig number (c) for each sequence, along with the accession number for the *S. suis* phage phi-SsuFJNP3_rum shown at right. Base pair numbers are displayed above the black bar at top. Green and yellow shaded bar below depicts sequence similarity with green indicating 100% identity and yellow indicating lower identity. Grey bar in between each aligned sequence region depicts sequence similarity between each sequence with grey indicating nucleotide positions with 100% identity. Blue arrows at end of grey bars indicate contig sequence continues and orange bars indicate end of contig sequence. AMR gene elements are listed below and indicated by pink (*vgb*) and yellow (*vat*). All other CDSs are indicated by light blue. Predicted incomplete CDSs are indicated by jagged ends. Accession number MN270260 is a complete nucleotide sequence from *S. suis* phage phi-SsuFJNP3_rum harbored by *S. suis* strain PJNP3, isolated from a pig.

Three isolates, 30428, 32052, and 40422, harbored the *vgb* gene, encoding virginiamycin B lyase and the *vat* gene, encoding streptogramin A O-acetyltransferase, which confers resistance to type B streptogramins through enzymatic inactivation. An initial BLASTN search revealed 100% sequence identity of the *vgb* gene from isolates 30428, 32052, and 40422 to the *vgb* gene of *Streptococcus* phage phi-SsuFJNP3_rum (accession no. MN270260; Figure 4; Supplementary Table 2). Further analysis revealed a high degree of nucleotide similarity between *Streptococcus* phage phi-SsuFJNP3_rum and isolates 30428, 32052, and 40422 spanning approximately 3.5 kb and including the *vgb* gene as well as three upstream CDSs. A 90.6% sequence identity was observed between the *vat* gene of *Streptococcus* phage phi-SsuFJNP3_rum and the *vat* gene harbored by isolates 30428, 32052, and 40422. Additionally, the *vat* gene harbored by *Streptococcus* phage phi-SsuFJNP3_rum was observed to be 21 bp longer than the *vat* gene from isolates 30428, 32052, and 40422 with 660 nucleotides, compared to 639 nucleotides. The nucleotide similarity between *Streptococcus* phage phi-SsuFJNP3_rum and isolates 30428, 32052, and 40422 drastically decreases downstream of the *vat* gene (Figure 4; Supplementary Table 2).

While 49 isolates exhibited phenotypic sulfonamide resistance, no acquired sulfonamide resistance genes, such as *sul1*, *sul2*, *sul3*, or *sul4* were found within any of the *S. suis* genomes. Given that mutations in the chromosomal *folP* gene have resulted in sulfonamide resistance, both the nucleotide and amino acid sequences in all *S. suis* draft genomes were analyzed and compared. Although sequence diversity within both nucleotide and amino acid sequences was observed, no correlation between the *folP* sequence and phenotypic sulfonamide resistance among the isolates was identified.

### AMR elements located within predicted MGEs

Whole-genome sequence data for all isolates in this study, and previously reported *S. suis* isolates (Nicholson et al., 2020), were screened *in silico* for MGEs. A total of 165 representative MGE sequences, including predicted plasmids, were identified with sequence lengths varying from 70 to 191,089 bp. Additionally, 17 contigs were identified as potential plasmids and ten IMEs varying in length from 47,963 to 69,374 bp (Supplementary Table 3). The 138 representative MGE sequences found throughout the analyzed genome sequences comprised 1,712 individual MGEs homologs, bringing the total number of MGE homologs, including predicted plasmids, to 1,739 (Supplementary Table 3). While some identified representative MGEs do not contain any coding sequence, most of the representative MGEs identified contained passenger genes coding for known functions, such as AMR elements (Supplementary Table 3). The frequency of individual representative MGE sequences counted across the isolates ranged from one to seventeen (Table 5; Supplementary Table 3). For example, a representative MGE sequence 11,006 bp in length harboring a passenger *tetO* AMR element was found in seventeen isolates and a representative MGE sequence 2,659 bp in length harboring a passenger *ermB* AMR element was found in seventeen isolates (Table 5; Supplementary Table 3). The AMR elements *ant(9)-Ia*, *ermB*, *ble*, *lnuB*, *lsaE*, and *tetO* were identified as passenger genes among the representative MGE sequences, with the highest frequency observed for *ermB*, identified among thirty representative MGEs, and *tetO*, identified among thirty-one representative MGEs (Table 5; Supplementary Table 3). Of the seventeen isolates harboring a contig identified as a potential plasmid, six harbored passenger AMR elements, including *tetM, tetO, ermB*, and/ or *ble* (Table 5; Supplementary Table 3). The IME harbored by isolates 29885, 30087, 30815, 40429, 40441, 40450, 40453, ISU1606, ISU2514, and ISU2614 contained *tetO*, *ermB*, and/or *ermL* passenger AMR elements (Table 5).

**Table 5:** AMR elements located within predicted MGEs.

Representative MGE ID	Length (bp)	MGE Type	AMR gene (locus_tag)	All isolates containing representative MGE ID
c1056_g113_s1056	17,428	IME	*ant(9)-Ia* (DK877_09725), *lnuB* (DK877_09745), *lsaE* (DK877_09750)	ISU2414
c1703_g122_s1703	32,277	IS Element, IME	*ermB* (DK875_06640)	ISU2514
c17669_g141_s5185	68,483	IME	*ermB* (DK876_03150), *tetO* (DK876_03195)	29932, 30114, 30428, 37904, 40418, 40424, 40444, 40469, 40534, 40535, ISU2714
c1769_g52_s1769	11,006	Unclassified Genomic Island	*tetO* (DK875_05055)	29898, 30068, 30087, 30118, 30413, 30807, 30815, 34114, 38529, 39631, 40429, 40432, 40437, 40450, 40456, 40464, ISU2514
c4221_g154_s4221	137,371	IS Element, Prophage, IME	*ermB* (A7J09_06245), *tetO* (A7J09_06320)	ISU2812
c4912_g121_s4912	17,439	Unclassified Genomic Island	*tetO* (HCB98_00820)	40525, ISU2614
c5870_g53_s5870	2,659	Islet	*ermB* (HCB73_07125)	30413, 38728, 40426, 40431, 40436, 40451, 40452, 40456, 40459, 40463, 40467, 40470, 40472, 40473, 40524, 40527, ISU2812
30,076_c44p	36,218	Plasmid	*tetM* (HCC69_11940)	20076
30,184_c12p	139,289	Plasmid	*tetM* (HCC65_07470), *ermB* (HCC65_07305)	30184
40,440_c12p	120,722	Plasmid	*tetM* (HCC27_08115)	40440
40,453_c14p	183,029	Plasmid	*tetO* (HCB76_10585), *ermB* (HCB76_10695)	40453
40,455_c25p	75,241	Plasmid	*tetM* (HCC15_08930), *ble* (HCC15_09190)	40455
40,462_c10p	76,433	Plasmid	*tetM* (HCC19_04160), *ble* (HCC19_04420)	40462
40,521_c12p	65,127	Plasmid	*ble* (HCC38_06105)	40521
29,885_c17:CH1.CH6	47,963	IME	*tetO* (HCC57_07895), *ermB* (HCC57_07985)	29885
30,087_c3:CH1.CH6	56,547	IME	*tetO* HCC51_01245, *ermB* (HCC51_01320)	30087
30,815_c5:CH1.CH6	63,420	IME	*tetO* (HCC63_02030)	30815
40,429_c10:CH1.CH6	57,085	IME	*tetO* (HCC08_07970), *ermB* (HCC08_07900)	40429
40,441_c7:CH1.CH6	61,082	IME	*tetO* (HCB75_06125), *ermB* (HCB75_06155)	40441
40,450_c12:CH1.CH6	56,547	IME	*tetO* (HCB88_05660), *ermB* (HCB88_05585)	40450
40,453_c14:CH1.CH6	58,064	IME	*tetO* (HCB76_10585), *ermB* (HCB76_10695)	40453
ISU1606:CH1.CH6	68,139	IME	*tetO* (DK235_04640), *ermB* (DK235_04660), *ermL* (DK235_04665)	ISU1606
ISU2514:CH1.CH6	61,633	IME	*tetO* (DK875_05055)	ISU2514
ISU2614:CH1.CH6	69,374	IME	*tetO* (DK237_05285), *ermB* (DK237_05150), *ermL* (DK237_05155)	ISU2614

### Virulence factor distribution

To examine genomic differences that may influence how *S. suis* isolates interact with their hosts and environment, we compared the nucleotide sequences of genes encoding virulence-associated factors, which were included in a comprehensive review (Fittipaldi et al., 2012). The percent identity for each gene was determined for each isolate relative to the P1/7 orthologue with the following exceptions: *epf, hylA*, *ofs, revS*, *stp*, *vraR* and *vraS*. These exceptions were based on choosing a reference gene sequence in which functional characterization had previously been reported using a different strain, or due to annotation of the P1/7 gene as a pseudogene. A complete list of all virulence-associated genes and the percent identity relative to the reference orthologue used for all *S. suis* isolates is provided in Supplementary Table 4.

The nucleotide sequence identity for all the virulence-associated genes analyzed ranged from 100 to 75.41% with an average of 96.08% (Supplementary Table 4). The lowest nucleotide sequence identity was observed for *sadP* (75.41%), *mrp* (78.98%), and *ofs* (80.56%) *and ideS* (83.86%). Genes *epf* and *nadR* were found in the draft genomes for only two isolates, 40424 and 40533, both CC1 isolates. *nadR* from these isolates were 100% identical to the P1/7 reference orthologue. The *epf* gene harbored by these isolates was 99.9% identical to the reference orthologue and did not encode the long form of extracellular protein factor (EF*), which can be expressed by some serotype 2 isolates and produce a high molecular weight variant of EF (>110 kDa; Galina et al., 1996; Gottschalk et al., 1998). Several of the virulence-associated genes analyzed were not found in the draft genomes for numerous isolates. Specifically, *revS* was not found or was absent in 93 isolates, *rgg* was absent in 92 isolates, and *neuB* was absent in 70 isolates (Supplementary Table 4).

Historically, the previously mentioned *epf* and *mrp* genes as well as the *sly* gene, encoding suilysin (a thiol-activated toxin hemolysin), were used to predict the virulence potential of *S. suis* isolates, particularly serotype 2 isolates obtained from European countries (Gottschalk et al., 1998; Wisselink et al., 2000; Fittipaldi et al., 2012). The *sly* gene was not found in the draft genomes for 54 isolates. However, the *sly* gene was highly conserved in the isolates that did harbor the gene, with an average 99.77% sequence identity (Supplementary Table 4). The *mrp* gene was not found in the draft genomes for 22 isolates and, as stated previously, a higher nucleotide sequence diversity was observed for *mrp,* with an average 78.97% sequence identity (Supplementary Table 4).

**Figure 5:**
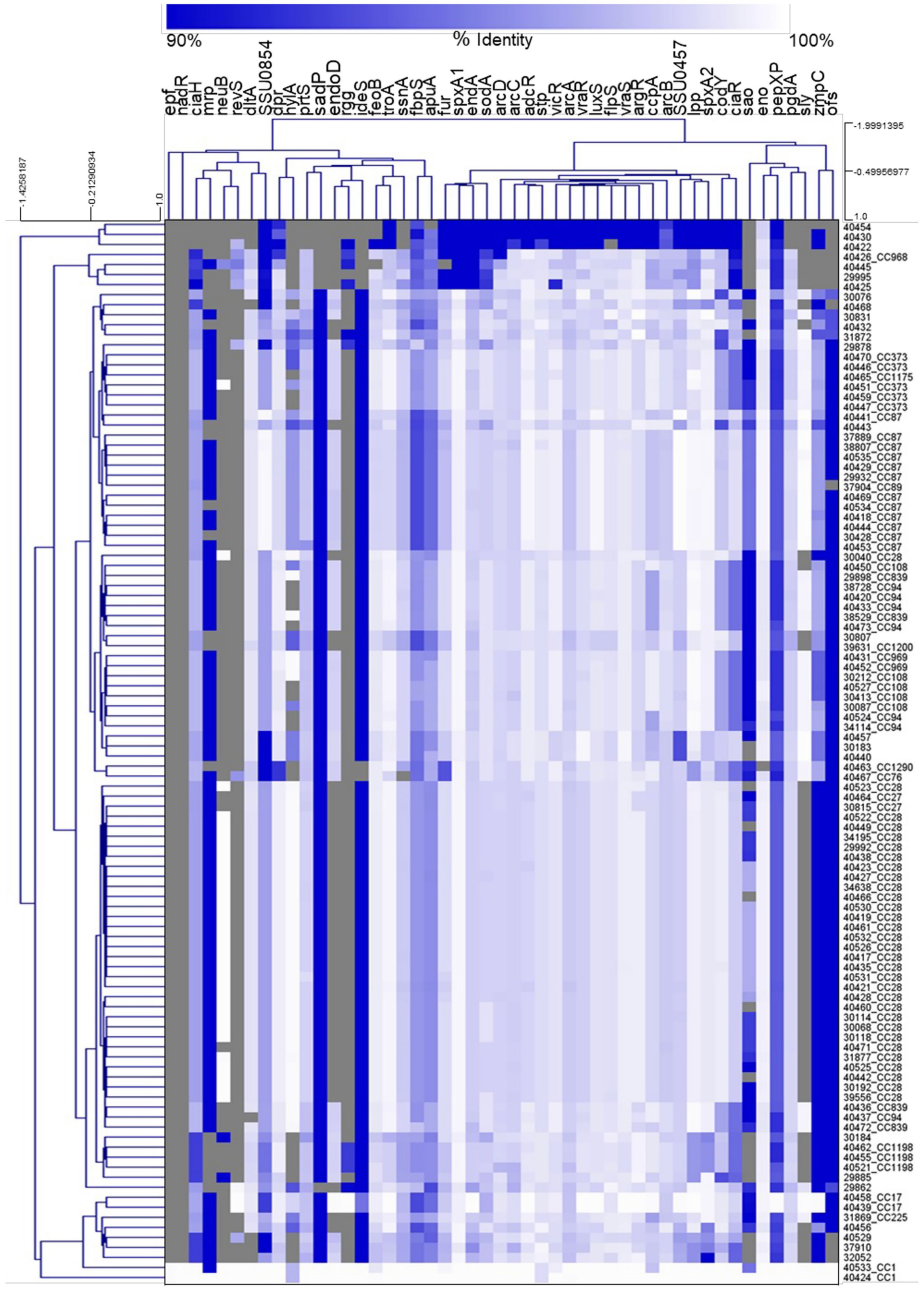
Hierarchical cluster heatmap displaying the relatedness of *S. suis* isolates based on the nucleotide percent identity of analyzed virulence genes. A distance matrix generated form the nucleotide percentage identity was converted into a heatmap and clustered by means of complete hierarchical clustering based on Pearson correlation distance for both genes and isolates. Gene names are provided at the top of the heat map and isolate names along with their corresponding CC are provided at the right side of heat map. Percent identity of analyzed genes (columns) from each isolate (rows) is represented using the color scale at top, while genes not present within an isolate are indicated by grey. Dendrograms are on the left side and on top of the heat map.

Hierarchical clustering analysis of the nucleotide percent identity for the virulence-associated genes was performed and revealed a correlation between CC and the presence of genes encoding known virulence factors, as well as the nucleotide identity among those genes (Figure 5). Specifically, all isolates from the CC87, CC1198, CC1, CC17, CC969, and CC27 were observed clustered or grouped together (Figure 5). Additionally, four out of the five CC108 isolates were observed clustered together, eleven out of the twelve CC87 isolates were observed clustered together, and twenty-nine of the thirty-one CC28 isolates were observed clustered together (Figure 5).

An *in silico* PCR was used to screen the isolate genomes sequence in this study, as well as previously reported *S. suis* genomes (Nicholson et al., 2020), employing primer sets described by Schmid et al. (2011). All isolates were negative for CH3/CH4 product, which is specific for the 89 K pathogenicity island (89 K PAI) carried by the extremely virulent *S. suis* isolate 05ZYH33 and other isolates responsible for outbreaks of severe human infections in China (Chen et al., 2007; Schmid et al., 2011). Twenty isolates were positive for *in silico* PCR products for both the CH1/CH2 primer set, specific for the upstream integration region, and the CH5/CH6 primer set, specific for the downstream integration region (Schmid et al., 2011). The products for both primer sets were found in ten isolates (29885, 30087, 30815, 40429, 40441, 40450, 40453, ISU1606, ISU2514, ISU2614; Table 5). Further analysis revealed MGEs, classified as IMEs, due to the absence of a gene encoding VirB4 ATPase (Johnson and Grossman, 2015), varying in length from 47,963 to 69,374 bp and harboring the 15-bp *att* site sequence (TTATTTAAGAGTAAC) at the 3′ end of *rplL* and intergenic region between the gene encoding a HAD family hydrolase and the gene encoding a tyrosine site-specific integrase. As previously mentioned, all IMEs harbored passenger AMR elements (Table 5).

To expand the search for genomic elements that could increase the capacity of *S. suis* to transmit, colonize, and/or cause disease in humans, the virulence factor database (VFDB) was employed to search the draft genome sequence data for all *S. suis* isolates for any predicted virulence-associated genes. A complete list of all the genes encoding predicted virulence-associated genes identified within the *S. suis* genomes is provided in Supplementary Table 4. No genes encoding known factors contributing to the capacity to transmit, colonize, and/or cause disease in humans were identified in any of the *S. suis* genomes (Supplementary Table 4). Thirty-three nonredundant gene products were identified, the majority of which included known *S. suis* virulence-associated genes and genes associated with capsule biosynthesis.

## Discussion

Given the importance of *S. suis* as a world-wide emerging human pathogen and potential AMR reservoir, the goals of the current study were to employ whole-genome sequencing (WGS) analysis to evaluate the ST and serotype distribution and screen these genomes for elements encoding AMR and any factors that could increase or contribute to the capacity of *S. suis* to transmit, colonize, and/or cause disease in humans. We identified a wide distribution of STs, which included forty-six different STs, with ST28 observed as the most prevalent, followed by ST87, ST94, and ST961. Similarly, a recent study evaluating *S. suis* isolates obtained within the U.S. also reported a wide distribution of STs along with a high prevalence of ST28 and ST94 isolates (Estrada et al., 2019). The same study also reported a high prevalence of ST1 isolates (Estrada et al., 2019). In contrast, only two isolates in this report were identified as ST1. ST1 isolates are highly prevalent in many areas of the world including Canada, South American, Europe, and Asia and are commonly associated with clinical systemic disease in both pigs and humans (Goyette-Desjardins et al., 2014; Auger et al., 2016; Lacouture et al., 2022). The low prevalence of ST1 isolates in this study could reflect the choice to avoid using clinical veterinary data as the strain inclusion criteria for this study. ST28 isolates have been reported as the most prevalent ST in the U.S., while ST25 have been reported as the most prevalent ST in Canada (Fittipaldi et al., 2011; Goyette-Desjardins et al., 2014). However, a recent study evaluating *S. suis* isolates obtained within the Quebec Canada reported ST1 as the most prevalent, followed by ST28, ST25, and ST94 (Lacouture et al., 2022). Like previous reports suggesting a low prevalence of ST25 in the U.S., no ST25 isolates were identified in this study. When the *S. suis* isolates were grouped into CCs, we found CC28 was the most prevalent, followed by CC87, and CC94. The overall distribution, along with the high prevalence of CC87 and low prevalence of CC1, reported here differs from recent studies evaluating *S. suis* isolates obtained within the U.S. and Canada, which reported a high prevalence of CC1 and low prevalence of CC87.

While a variety of virulence factors have been described for *S. suis*, CPS is by far considered the most important (Charland et al., 1998; Fittipaldi et al., 2012; Roy et al., 2015; Zhao et al., 2015; Segura et al., 2017). Worldwide, the predominant *S. suis* serotypes isolated from clinical cases in pigs are, in decreasing order, serotypes 2, 9, 3, 1/2, and 7 (Goyette-Desjardins et al., 2014; Segura et al., 2017). Serotype 2 isolates are the most frequently isolated and associated with *S. suis* clinical disease cases in both swine and humans globally, and thus have been historically considered the most virulent and zoonotic (Fittipaldi et al., 2012; Goyette-Desjardins et al., 2014). In North America, serotypes 2 and 3 have been reported as the most prevalent serotypes isolated from clinical pig cases, followed by serotypes 1/2, 8 and 7 (Gottschalk et al., 2007; Messier et al., 2008; Fittipaldi et al., 2009; Goyette-Desjardins et al., 2014). Recently, serotypes ½, followed by 7, 3 and then 2 have been reported as the most prevalent serotypes in the U.S. (Estrada et al., 2019). We identified 23 different serotypes with serotype 2 observed as the most prevalent, followed by serotype 3, and 8. Focusing on the serotype 2 isolates, nineteen of these were ST28, five were ST961, three were ST1180, and one was ST1199. The classical epf+/mrp+/sly+ genotype typically associated with serotype 2 isolates was not observed among the isolates in this study. All of the serotype 2 isolates harbored the *mrp* gene, however, none of these isolates harbored the *epf* gene, and the one ST119 isolate was the only serotype 2 isolate that harbored the *sly* gene.

A relatively high phenotypic AMR prevalence was observed, given that half of the isolates exhibited resistance to three out of the eight antibiotic classes tested. The highest frequencies of resistance were observed for tetracycline, followed by MLSb, and sulfonamide antibiotic classes. Conversely, a low frequency of resistance was observed for β-lactams. The overall frequency and distribution of genetic determinants underlying AMR reflected the phenotypic AMR prevalence. A notable exception was for phenotypic sulfonamide resistance where no genetic determinant was able to be identified. The most prevalent AMR element found among the isolates was *tetO*, followed by *ermB, lsaE*, *lnuB*, *ant(9)-Ia*, and *ant(6)-Ia*. While differences in MICs between countries have been described, high prevalence rates for macrolide and tetracycline resistance, along with low prevalence rates for β-lactam resistance have been previously reported (Gurung et al., 2015; Aradanas et al., 2021; Dechene-Tempier et al., 2021; Hadjirin et al., 2021; Ma et al., 2021; Matiasovic et al., 2021; Cucco et al., 2022). This has been a global trend for several decades now, and the genetic basis for these resistances has been extensively studied (Varela et al., 2013; Gurung et al., 2015; Seitz et al., 2016; Dechene-Tempier et al., 2021). Resistance to MLSb has been reported to due to the occurrence of *ermB*, while resistance to tetracycline has been reported to be mainly associated with the presence of *tetO* and, to a lesser extent, *tetM* and the mosaic gene *tet* (O/W/32/O; Palmieri et al., 2011b; Varela et al., 2013; Gurung et al., 2015; Seitz et al., 2016; Dechene-Tempier et al., 2021). All of which were identified among the *S. suis* isolates in this study.

Three unexpected or novel AMR elements were identified among the *S. suis* isolates in this study. The *sat4* was co-located with the *ant(6)-Ia* and *aph(3′)-IIIa* genes in five isolates that shared high sequence similarity to the *E. faecium* multidrug resistance conjugative plasmid and the *E. rhusiopathiae* integrative conjugative element. To our knowledge, there has only been one previous report of *S. suis* harboring the *sat4* gene (Palmieri et al., 2011a). No sequence similarity was observed between this previously described region and the region within the five isolates harboring the *sat4* gene in this study. The *vgb* gene, encoding virginiamycin B lyase, and the *vat* gene, encoding streptogramin A O-acetyltransferase, conferring resistance to type B streptogramins was found in three isolates. The region harboring the *vgb* and *vat* genes from these isolates shared high sequence similarity to a *Streptococcus* phage phi-SsuFJNP3_rum. Genbank records indicate that phage phi-SsuFJNP3_rum was obtained from *S. suis* isolate FJNP3. Outside of this Genbank submission record, this is the first report of *S. suis* harboring the *vgb* and *vat* genes.

When the draft genomes were searched for MGEs a total of individual 1,739 MGEs, representing a wide diversity of MGE types were identified. For example, IMEs harboring passenger AMR elements as well as the 15-bp *att* site sequence (TTATTTAAGAGTAAC) at the 3′ end of *rplL,* which is highly conserved in *Streptococcus* spp. and a common site for recombination and/or insertion (Huang et al., 2016a,b). The passenger AMR elements located within all MGE sequences, including predicted plasmids were *ant(9)-Ia*, *ble*, *lnuB*, *lsaE*, *ermB*, *ermL, tetM*, and *tetO*, with the highest frequency observed for *ermB* and *tetO*. Previous studies have demonstrated the substantial role MGEs serve in the horizontal transfer of AMR elements (Huang et al., 2016b; Libante et al., 2019). Collectively, the data reported here indicates the capacity of *S. suis* isolates to gain and lose MGEs encoding AMR elements and thus supports concerns regarding the capacity of these isolates to disseminate AMR.

As previously mentioned, the classical *epf*+/*mrp*+/*sly* + genotype typically associated with serotype 2 isolates was not observed among the isolates in this study. Only two isolates 40424 and 40533, were found to harbor all three classical virulence-associated genes *epf*, *mrp*, and *sly*, as well as *nadR*. Both 40424 and 40533 were isolated from the U.S. midwest (IN and IL) and were ST1, serotype 14, and CC1. Hierarchical clustering of the nucleotide percent identity for the virulence-associated genes revealed a correlation between CC and the presence of genes encoding known virulence factors. While similar correlations between virulence-associated genes and CCs or lineage has been previously described (Fittipaldi et al., 2011; Dong et al., 2017; Nicholson et al., 2020; Estrada et al., 2021; Kerdsin et al., 2021; Estrada et al., 2022), very few virulence factors have been experimentally verified in swine challenge studies. Additionally, no clear correlation between the capacity to cause disease in swine and the presence of genes encoding known virulence factors, and the nucleotide identity among those genes, has been reported (Nicholson et al., 2020).

Notably, no genes encoding known factors contributing to the capacity to transmit, colonize, and/or cause disease in humans were identified in any of the *S. suis* genomes. This includes the 89 K pathogenicity island (89 K PAI) carried by virulent *S. suis* isolates responsible for outbreaks of severe human infections in China. Collectively, the broad inclusion of *S. suis* isolates obtained from within the U.S. included in this study provides a comprehensive evaluation of the genetic diversity among U.S. *S. suis* isolates to serve as a blueprint for determining any potential risks associated with occupational exposure to these bacteria, while also providing important data to address public concerns.

## Data availability statement

The datasets presented in this study can be found in online repositories. The names of the repository/repositories and accession number (s) can be found in the article/Supplementary material.

## Author contributions

TN conceived and designed the experiments. TN and DB performed the experiments, analyzed the data, contributed reagents/materials/analysis tools, and wrote the paper. All authors gave approval of the final version to be published and agreed to be accountable for all aspects of the work.

## Funding

Funding was provided by the United States Department of Agriculture, Agriculture Research Service project number 5030-32000-119-00-D. This research used resources provided by the SCINet project of the USDA Agricultural Research Service, ARS project number 0500-00093-001-00-D. Mention of trade names or commercial products in this article is solely for the purpose of providing specific information and does not imply recommendation or endorsement by the USDA, DOE, or ORISE. USDA is an equal opportunity provider and employer.

## Conflict of interest

The authors declare that the research was conducted in the absence of any commercial or financial relationships that could be construed as a potential conflict of interest.

## Publisher’s note

All claims expressed in this article are solely those of the authors and do not necessarily represent those of their affiliated organizations, or those of the publisher, the editors and the reviewers. Any product that may be evaluated in this article, or claim that may be made by its manufacturer, is not guaranteed or endorsed by the publisher.

## Footnotes

^1^ https://www.bioinformatics.babraham.ac.uk/projects/fastqc/

^2^ https://github.com/lh3/seqtk

^3^ http://mira-assembler.sourceforge.net/docs/DefinitiveGuideToMIRA.html

^4^https://pubmlst.org/organisms/streptococcus-suis, accessed 17 February 2022

^5^ http://github.com/tseemann/abricate
